# Emotion recognition in generalized anxiety disorder, panic disorder, body dysmorphic disorder, skin picking disorder, trichotillomania, and posttraumatic stress disorder: a systematic review

**DOI:** 10.3389/fpsyg.2025.1486765

**Published:** 2025-07-04

**Authors:** Grace L. Wheeler, Shari A. Steinman

**Affiliations:** ^1^Department of Psychology, West Virginia University, Morgantown, VA, United States; ^2^Department of Psychological Science, University of Vermont, Burlington, VT, United States

**Keywords:** emotion recognition, anxiety disorders, obsessive-compulsive disorders, trauma-and stressor-related disorders, anxiety and related disorders

## Abstract

This systematic review evaluates patterns of emotion recognition in anxiety, obsessive-compulsive and related, and trauma- and stressor- related disorders (generalized anxiety disorder, panic disorder, body dysmorphic disorder, skin picking disorder, trichotillomania, and posttraumatic stress disorder). A comprehensive literature search identified 15 studies. Emotion recognition patterns were reviewed for each disorder. After considering strengths and weaknesses of individual studies and the literature as a whole, trends across disorders support a decreased recognition of sadness and an anger interpretation bias (i.e., tendency to see anger when other emotions are displayed). Conclusions are limited by a lack of covariate analyses and task validation. Future studies should investigate whether comorbid depression, transdiagnostic factors, and/or clinical severity may better explain variability in emotion recognition deficits.

## Emotion recognition

Emotion recognition is the ability to accurately identify another's emotion expression (Cai et al., 2012). A burgeoning body of research demonstrates emotion recognition deficits in many clinical patient populations (Kessler et al., 2007). Emotion recognition is linked to the maintenance and etiology of disorders categorized by fears of negative evaluations, such as anxiety, obsessive compulsive and related, and trauma and stressor related disorders (Buhlmann et al., 2006). Difficulties in recognition, leads to perceived negative feedback, increasing distress, and thereby exacerbating symptom experience. In the current systematic review, we review patterns of emotion recognition across anxiety, obsessive-compulsive, and trauma- and stressor- related disorders.

Emotion recognition tasks in which facial stimuli are presented for identification is a leading methodology to examine recognition biases (Buhlmann et al., 2006). Such stimuli allow for ecologically valid procedures to investigate emotion processing, identification, and misinterpretation (Buhlmann et al., 2006). Given that emotion vocabulary can be culturally defined or personally influenced, current research relies on tasks in which participants identify stimuli (e.g., photo of an actor displaying the emotion) of basic emotions (i.e., likely to have evolutionary-relevance and therefore be cross-culturally understood) and undergo a forced-choice procedure of potential emotional labels (Young et al., 2002). With such parameters, emotion recognition tasks can be easily compared across stimuli and methodologies.

## Previous reviews of emotion recognition and clinical disorders

Previous meta-analyses were found for emotion recognition patterns in social anxiety disorder (SAD) and obsessive-compulsive disorder (OCD), and consequently, these disorders are not included in the current review. Lacombe and colleagues concluded SAD was linked to difficulty recognizing emotional expressions and that comorbidity further impairs happiness recognition (Lacombe et al., 2023). Daros et al. (2014) found OCD was linked to worse accuracy in overall recognition and overall negative emotion recognition. The results for overall negative emotion recognition were driven by decreased recognition of disgust and anger.

Although Bottinelli et al. (2021) performed a mini-review of emotion recognition studies in panic disorder (PD), the authors did not include sufficient examination into study quality, methodology, and statistical techniques. Consequently, PD will therefore be included in the current systematic review.

## Aim of presented review

Emotion recognition research has been conducted within anxiety and related disorders (obsessive-compulsive and trauma- and stressor- related disorders; Aydin et al., 2022; Bell et al., 2017; Buhlmann et al., 2004; Cai et al., 2012; Palm et al., 2011). While there is sufficient synthesis of patterns in SAD and OCD, a gap remains in understanding available trends within generalized anxiety disorder (GAD), PD, body dysmorphic disorder (BDD), skin picking disorder (SPD), trichotillomania (TTM), and posttraumatic stress disorder (PTSD). Thus, the objective of the current review is to identify and differentiate patterns of emotion recognition among anxiety, obsessive-compulsive and related disorders (OCRDs), and trauma/stressor related disorders. The current review has been reported within standards set by the Preferred Reporting Items for Systematic Reviews and Meta-Analyses (PRISMA) guidelines (Page et al., 2021).

## Methods

### Eligibility criteria and information sources

There is no registered review protocol for this systematic review. A comprehensive literature search was conducted to ascertain relevant studies published through June 1st, 2024. The first author conducted the literature search between approximately June 1st, 2023 and June 1st, 2024. The following databases were used in the current review: PsychINFO, PubMed, and Google Scholar. Furthermore, an examination of reference lists of initially selected articles was conducted to ensure saturation of articles in the field. All articles included (*n* = 15) were peer-reviewed, published articles. See Appendix A for search terms.

Eligible studies were required to include one of the following terms in the title or abstract: “emotion recognition,” “emotion perception,” or “emotion identification.” In initial cursory reviews of the field, such terms were found to be used interchangeably. Studies must have included the appropriate clinical disorder in the title or abstract (e.g., “generalized anxiety disorder” or “GAD”) as defined by the DSM-5 (American Psychiatric Association, 2013). Literature search was conducted for all clinical diagnoses characterized as anxiety, obsessive-compulsive and related, and trauma- and stressor-related disorders per DSM-5 classifications (American Psychiatric Association, 2013).

Eligible studies were required to include an adult sample, a clinical group with a relevant diagnosed disorder (either anxiety, obsessive-compulsive and related, or trauma- and stressor-related), and a control group as defined as participants without the relevant diagnosis. Participants needed to label each emotional stimulus. Studies must have included data analysis on accuracy per emotion, rather than one aggregate score. This criterion was included to aid in synthesis and pattern determination for particular emotions. Exclusion criteria included sole use of two-dimensional (2-D) line drawing for emotion stimuli and use of an emotional prime before task. Sole reliance on 2-D emotion stimuli was excluded given the lack of ecological validity; results and conclusions based on 2-D drawings are less transferrable to real-world situations and symptom experiences. Emotional primes impact behavioral results and therefore results do not determine baseline patterns and cannot be synthesized with the broader field. Following inclusion and exclusion criteria, the current systematic review evaluates the following clinical diagnoses: generalized anxiety disorder (excessive, uncontrollable worry; Palm et al., 2011), panic disorder (sudden, unexpected episodes of fear with physiological symptoms such as increased heart rate and sweating; Cai et al., 2012), body dysmorphic disorder (preoccupation with perceived flaws in appearance; Buhlmann et al., 2004), skin-picking disorder (repetitive and compulsive skin picking; Aydin et al., 2022), trichotillomania (repetitive and compulsive hair pulling; Aydin et al., 2022), and posttraumatic stress disorder (clinical distress resulting from trauma; Bell et al., 2017).

### Data extraction and evaluation

After review of potential articles, pertinent data was obtained by the first author. Study quality was determined by evaluation of descriptive, methodological, and analytical data available. Data was obtained regarding sample characteristics, recruitment sources, task methodology and characteristics, task validation, and statistical analyses. Study limitations, power, and effect sizes were reviewed if available. Unreported power and effect sizes were calculated by the first author if sufficient information was provided in text. Extracted data were evaluated to synthesize emotion recognition patterns within disorders.

## Results

### Study selection

**Figure 1:**
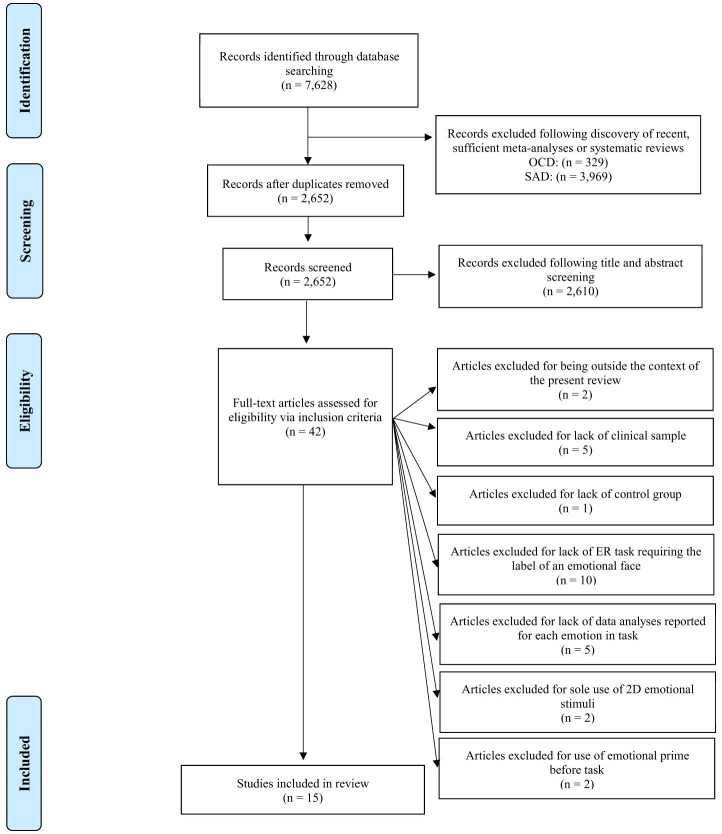
Preferred reporting items for systematic reviews and meta-analyses (PRISMA) flow diagram (Page et al., 2021).

The first author's preliminary search with the provided keywords produced a total of 7,628 abstracts from PsychINFO (6,012), PubMed (1,556), and Google Scholar (60). As recent, sufficient meta-analyses and systematic reviews were discovered for SAD and OCD, articles on these disorders were removed from the current review (Daros et al., 2014; Lacombe et al., 2023). Of the resultant articles, 678 duplicates were removed, and 2,610 articles were removed based on a screening of titles and abstracts. The remaining 42 articles were further screen through full-text reading for eligibility. Of those, two articles were determined to be unrelated to the current review (e.g., related to neurological imaging). The outstanding 40 articles were then reviewed in line with review inclusion criteria. Articles were then culled if they did not have a clinical group with diagnosed anxiety, obsessive-compulsive and related, or trauma- and stressor-related disorder (*n* = 5), a control group (*n* = 1), an emotion recognition task with labeling (*n* = 10), data analysis provided on accuracy based on emotion (*n* = 5), and three-dimensional emotion stimuli (*n* = 2). Studies were also excluded for emotional prime use before task (*n* = 2). Thus, a final selection of 15 articles was included in the present review (see Figure 1 for the PRISMA flow diagram of study selection; Page et al., 2021).

### Study characteristics

The 15 articles identified included a total of 401 relevant clinical patients, 400 non-clinical patients studied, and 206 participants in an additional comparison group (i.e., another clinical group or had a relevant shared experience to the clinical group). Studies were conducted in 10 countries. Seven studies reported comorbidities in their samples and four studies excluded presence of particular comorbidities. All studies used within-subject research designs and nine reported randomization of stimuli presentation. Thirteen studies used static stimuli and two studies used video stimuli. Five of the included studies used emotional stimuli that was morphed via computerized program to display differing emotion intensities. Anger was tested in all studies (*n* = 15), then happiness, sadness, and fear (*n* = 14), disgust (*n* = 13), surprise (*n* =12), neutrality (*n* = 11), contempt (*n* = 2), pride (*n* = 1), and embarrassment (*n* = 1). All study designs were cross-sectional. To review additional study characteristics, see Tables 1, 2.

**Table 1:** Demographic information and participant characteristics.

**References**	**Study groups**	**Clinical *N***	**Control *N***	**Additional group *N***	**Sample (country)**	**Gender (% female)**	**Mean age (years)**	**Medication Tx (*N*)**	**Clinical recruitment source**
Palm et al. (2011)	GAD, C	15	16	NA	United Kingdom	100%	34	Yes (3)	Community
Cai et al. (2012)	PD, C	21	34	NA	China	56.36%	26.75	NR	Outpatient
Kessler et al. (2007)	PD, C	37	43	NA	Germany	73.75%	37.05	Yes	Outpatient
Reinecke et al. (2011)	PD, C	23	22	NA	United Kingdom	71.47%	27.43	Excluded	Community
Wang et al. (2013)	PD, C	24	20	NA	South Korea	54.54%	47.07	Yes	Outpatient
Bell et al. (2017)	PTSD, Earthquake-Exposed, C	28	50	89	New Zealand	65.87%	45.40	Yes	Outpatient
Pfaltz et al. (2019)	PTSD, Trauma-experienced, C	39	35	44	Switzerland	44.92%	37.26	Excluded antipsychotics, benzodiazepines, tricyclic anti-depressants	Outpatient and community
Poljac et al. (2011)	PTSD, C	20	20	NA	Bosnia and Herzegovina	0%	41.85	NR	Self-help group
Buhlmann et al. (2004)	BDD, OCD, C	20	20	20	United Stated	61.67%	32.20	NR	Outpatient
Buhlmann et al. (2006)	BDD, C	18	18	NA	United Stated	80.56%	28.95	NR	Outpatient
Buhlmann et al. (2011)	BDD, Dermatological-conditions, C	34	34	34	Germany	65.63%	31.67	NR	Community
Grace et al. (2019)	BDD, C	19	21	NA	Australia	67.5%	33.01	Yes (14)	Outpatient
Jefferies et al. (2012)	BDD, C	12	16	NA	United Kingdom	60.71%	33.35	Yes (7)	Outpatient
Toh et al. (2015)	BDD, OCD, C	21	21	19	Australia	70.49%	35.62	Yes (20)	Outpatient
Aydin et al. (2022)	TTM, SPD, C	40 (SPD) 30 (TTM)	30	NA	Turkey	84%	29.51	NR	NR

GAD, Generalized Anxiety Disorder; PD, Panic Disorder; PTSD, Posttraumatic Stress Disorder; BDD, Body Dysmorphic Disorder; OCD, Obsessive-Compulsive Disorder; TTM, Trichotillomania; SPD, Skin Picking Disorder; C, control group; NR, not reported; Community recruitment sources included online ads, local press, university emails, and flyers.

**Table 2:** Emotion recognition task information.

**References**	**Disorder of interest**	**Emotions tested**	**Type of stimuli**	**Stimuli (ms)**	**Study design**	**Label procedure**	**Trials**
Palm et al. (2011)	GAD	Anger, happiness, sadness, fear, disgust, neutrality, surprise	Natural photos and morphed images	500	Boxcar	During	62
Cai et al. (2012)	PD	Anger, happiness, sadness, fear, disgust, surprise, contempt	Natural photos	5,000	Randomized	NR	56
Kessler et al. (2007)	PD	Anger, happiness, sadness, fear, disgust, surprise	Natural photos	300	NR	After	84
Reinecke et al. (2011)	PD	Anger, happiness, sadness, fear, disgust, surprise	Natural photos and morphed images	500	NR	During	250
Wang et al. (2013)	PD	Anger, happiness, sadness, fear, neutrality	Natural photos and morphed images	500	Randomized	NR	170
Bell et al. (2017)	PTSD	Anger, happiness, sadness, fear, disgust, neutrality	Natural photos and morphed images	500	Randomized	NR	150 (current) or 96 (previous)^a^
Pfaltz et al. (2019)	PTSD	Anger, happiness, sadness, fear, disgust, neutrality, surprise, pride, contempt, embarrassment	Natural videos	1,000	NR	After	300
Poljac et al. (2011)	PTSD	Anger, happiness, sadness, fear, disgust, neutrality, surprise	Videos including natural photos and morphed images	500–2,000	Stratified-random	During	216
Buhlmann et al. (2004)	BDD	Anger, happiness, sadness, scared, disgust, neutrality, surprise	Natural photos	15,000	Randomized (clinical); yoked (control)	NR	42
Buhlmann et al. (2006)	BDD	Anger, disgust, neutrality, surprise	Natural photos	NR	NR	NR	24
Buhlmann et al. (2011)	BDD	Anger, happiness, sadness, scared, disgust, neutrality, surprise	Natural photos	NR	NR	During	28
Grace et al. (2019)	BDD	Anger, happiness, sadness, fear, neutrality	Natural photos	200 or 2,000	Counter-balanced	During and after	200
Jefferies et al. (2012)	BDD	Anger, happiness, sadness, fear, disgust, surprise	Natural photos	5,000	Randomized	During	60
Toh et al. (2015)	BDD	Anger, happiness, sadness, fear, disgust, neutrality, surprise	Natural photos	8,000	Pseudo-randomized	After	84
Aydin et al. (2022)	TTM/SPD	Anger, happiness, sadness, fear, disgust, neutrality, surprise	Natural photos	Unrestricted^b^	NR	During	56

GAD, Generalized Anxiety Disorder; PD, Panic Disorder; PTSD, Posttraumatic Stress Disorder; BDD, Body Dysmorphic Disorder; OCD, Obsessive-Compulsive Disorder; TTM, Trichotillomania; SPD, Skin Picking Disorder; NR, not reported.

a In Bell et al. (2017), the HC group participated in a slightly different FER task in which they were presented with fewer faces for each emotion and fewer neutral faces, leading to total fewer trials. Analyses were conducted only on the 96 shared stimuli.

b In Aydin et al. (2022), participants were told they would only have 10 seconds to label the emotional stimulus, but responses were not actually timed/restricted by the study team.

### Summary of the literature

See Tables 1–3 for a summarization of sample characteristics, emotion recognition task information, and findings, respectively. Results not related to baseline emotion recognition (e.g., eye tracking, dot-probe, emotion training) will not be reviewed as they are outside the scope of the current review.

**Table 3:** Summary of study findings.

**References**	**Study groups**	**Comorbid disorders clinical group (*N*)**	**Covariate analyses**	**Findings**
Palm et al. (2011)	GAD, C	Past MDD (8); Anxiety disorders (10); 1 AUD (remission)	None	GAD group (M = 50) had significantly worse recognition of sadness than control group (M = 61, *p* < 0.05).
Cai et al. (2012)	PD, C	Exclusions: Axis 1 disorder, substance abuse history	None	PD group had significantly worse recognition for disgust (PD: M = 0.57; Control: M = 0.77, *p* = 0.03) and fear (PD: M = 0.47; Control: M = 0.67, *p* = 0.01), and better recognition of surprise (PD: M = 0.91; Control: M = 0.79, *p* = 0.01) than control group.
Kessler et al. (2007)	PD, C	Exclusions: other psychiatric disorders	Depression, anxiety	PD group had significantly worse recognition for sadness (PD: M = 4.8; Control: M = 6.5, *p* = 0.006), anger (PD: M = 6.5; Control: M = 8.0, *p* < 0.001), and overall emotion (PD: M = 37.5; Control: M = 45.4, *p* = 0.003) recognition than control group. When depression and anxiety were controlled for, results were no longer significant. PD group significantly misinterpreted other emotions as anger (*p* = 0.03).
Reinecke et al. (2011)	PD, C	GAD (3); OCD (2); SAD (1); Anorexia nervosa (1); MDD (1); Dysthymia (1)	None	PD group had significantly better recognition for sadness than control group (*p* < 0.05). No group differences in misinterpretations.
Wang et al. (2013)	PD, C	NR	Medication	PD group had significantly better accuracy for overall emotion recognition (PD: M = 51.04; Control: M = 45.38, *p* = 0.041). Participants on Escitalopram and Paroxetine did not differ in emotion recognition abilities; there were no significant correlations between medication dosage or duration with emotion recognition accuracy.
Bell et al. (2017)	PTSD, Earthquake-Exposed (EE), Non-exposed control (HC)	NR	Depression, anxiety	PTSD and EE groups had significant better recognition for neutral (*p* = 0.003), angry (*p* < 0.001), happy (*p* < 0.001), sad (*p* < 0.001), fearful (*p* < 0.001), and disgust (*p* < 0.001) faces (all emotions presented) compared to HC group. PTSD and EE groups misinterpreted neutral expressions as angry (*p* < 0.001) and disgusted (*p* = 0.003). HC group misinterpreted neutral expressions as happy (*p* < 0.001). When depression and anxiety were included as covariates, there was no longer a significant result for decreased sadness recognition in PTSD group compared to EE group.
Pfaltz et al. (2019)	PTSD, Trauma-experienced, No-trauma controls (HC)	NR	None	There was no difference in recognition accuracy or misinterpretation between groups. Higher levels of childhood sexual abuse were linked to worse accuracy for neutral faces (*p* = 0.001). Higher levels of childhood neglect, sexual abuse, and emotional abuse were linked to misinterpretation of neutrality as contempt (neglect: *p* = 0.006; sexual abuse: *p* = 0.003, emotional abuse: *p* = 0.014) and higher levels of childhood sexual abuse and neglect were linked to misinterpretation of neutrality as anger (sexual abuse: *p* = 0.002, neglect: *p* = 0.003).
Poljac et al. (2011)	PTSD, C	NR; Exclusions: psychosis, bipolar disorder, major mood or anxiety disorder, substance abuse	Depression	PTSD group had significantly worse recognition of fear (*p* < 0.01) and sadness (*p* = 0.05) compared to the control group. Depression did not influence results.
Buhlmann et al. (2004)	BDD, OCD, C	NR	None	BDD group had significantly worse recognition of neutrality (BDD: M = 4.8; Control: M = 5.7, *p* < 0.05), disgust (BDD: M = 4.8; Control: M = 5.7, *p* < 0.05), and overall emotion (BDD: M = 35.4; Control: M = 38.0, *p* < 0.05) than control group. BDD group significantly misinterpreted disgust as anger (*p* = 0.01) and more faces as anger than control group (*p* = 0.002). There were no group differences between BDD and OCD groups.
Buhlmann et al. (2006)	BDD, C	SAD (6); MDD (4); OCD (5). Anorexia nervosa (3); GAD (3); Specific phobia (2); 1 Bulimia nervosa (1); Dysthymia (1); PD (1); PTSD (1)	Subgroup analyses without SAD and MDD	BDD group was significantly less accurate for overall recognition (BDD: M = 16.8; Control: M = 19.3, *p* = 0.007) and recognition of neutral faces in self-referent scenarios (BDD: M = 3.8; Control: M = 5.3, *p* = 0.003). BDD misinterpreted more neutral faces as contempt (*p* = 0.001) and as anger (*p* = 0.04). When subgroup analyses were completed without individuals with SAD and MDD, respectively, it was found SAD did not influence results and MDD may have influenced results.
Buhlmann et al. (2011)	BDD, Dermatological-conditions (DC), C	SAD (11); MDD (10); Dysthymia (6); Specific phobia (5); alcohol dependence (2); Bulimia nervosa (2); OCD (2); PD w/o agoraphobia (1); PTSD (1); substance dependence (1)	None	BDD group (BDD: M = 3.6) had significantly worse recognition of neutral faces than the DC (DC: M = 3.9, *p* = 0.46) and control groups (Control: M = 4, *p* = 0.02).
Grace et al. (2019)	BDD, C	MDD or dysthymia (4); Agoraphobia or SAD (5)	OCD symptoms, depression, anxiety	BDD group had significantly worse recognition of anger (BDD: M = 74.12; Control: M = 80.50, *p* = 0.003), sadness (BDD: M = 77.65; Control: M = 89.50, *p* = 0.01), neutrality (BDD: M = 49.71; Control: M = 69.29, *p* = 0.024), and overall emotion recognition than control group. No influence of OCD symptoms, depression, or anxiety.
Jefferies et al. (2012)	BDD, C	OCD, MDD, OCPD, SAD, Tourette's, and/or Gender Identity Disorder^a^	Anxiety and depression	BDD group had significantly worse recognition of fear (BDD: M = 5.08; Control: M = 7.69, *p* = 0.04) than control group. BDD recognized *no-threat* emotions significantly better than *threat* emotions (*p* < 0.001). Anxiety and depression did not affect results.
Toh et al. (2015)	BDD, OCD, C	MDD (9); SAD (7); OCD (5)	None	BDD group had significantly worse overall emotion recognition compared to control and OCD groups. There was not a significant interaction between group and emotion. BDD group misinterpreted more faces as angry relative to control and OCD groups (*p* = 0.004).
Aydin et al. (2022)	TTM, SPD, C	NR; exclusions: bipolar, psychosis, suicide risk, alcohol/substance abuse	Alexithymia, depression, anxiety	SPD group had significantly worse recognition of disgust (SPD: M = 3.98; Control: M = 5.87, *p* = 0.003) and overall emotion recognition (SPD: M = 35.8; Control: M = 41.4, *p* = 0.004) compared to control group.

GAD, Generalized Anxiety Disorder; PD, Panic Disorder; PTSD, Posttraumatic Stress Disorder; BDD, Body Dysmorphic Disorder; OCD, Obsessive-Compulsive Disorder; TTM, Trichotillomania; SPD, Skin Picking Disorder; C, control group; NR, not reported.

a All clinical participants in Jefferies et al. (2012) had at least one comorbid disorder.

#### Generalized anxiety disorder (*n* = 1)

In investigating patterns within GAD (*n* = 15) and controls (*n* = 16), Palm et al. (2011)^1^ tested recognition of anger, disgust, fear, happiness, sadness, and surprise, as well as neutral stimuli. Stimuli were presented at 10 different intensities (in 10% increments) and in a boxcar randomization design (alternating neutral and emotional faces). Results demonstrated GAD participants were less accurate in recognizing sadness than controls.

#### Panic disorder (*n* = 4)

Cai et al. (2012) looked at recognition of anger, contempt, disgust, fear, happiness, sadness, and surprise in PD without agoraphobia (*n* = 21) and a control group (*n* = 34). The PD group had significantly lower accuracy for disgust and fear but were more accurate for surprise. There were no significant group differences in evaluating intensity of the emotion expressed.

Kessler et al. (2007) studied individuals with PD without agoraphobia (*n* = 37) compared to controls (*n* = 43) in recognizing fear, anger, sadness, happiness, and disgust. The control group was receiving treatment for peripheral neurological disorders (e.g., nerve lesions due to lumbar disc hernia). The PD group had impaired recognition in sadness and anger, and overall worse emotion recognition. However, when controlling for depression and anxiety scores, the effect of group was no longer significant for recognition accuracy. The PD group also had a tendency to misinterpret other emotions as anger.

Reinecke et al. (2011) also tested a PD clinical group (*n* = 23) compared to a control sample (*n* = 22) on recognition of anger, disgust, fear, sadness, happiness, or surprise. Within the PD group, eight participants had agoraphobia and 15 did not. The PD group had enhanced recognition of sadness compared to the control group. There was also no difference in misinterpretation of emotional expression between groups.

Wang et al. (2013) examined patterns within PD (*n* = 24) and controls (*n* = 20) on the recognition of happiness, sadness, anger, and fear. The PD group had significantly better recognition accuracy than control group. There was no significant group difference for each specific emotion, but there were non-significant trends for an enhanced recognition of fear. Analyses found participants on Escitalopram and Paroxetine did not differ in recognition abilities and there were no significant correlations between medication dosage or duration of medication with emotion recognition accuracy.

#### Posttraumatic stress disorder (*n* = 3)

Bell et al. (2017) compared individuals with PTSD caused by exposure to an earthquake (PTSD; *n* = 28), individuals who had exposure to an earthquake but did not develop PTSD (EE; *n* = 89), and non-exposed controls (HC; *n* = 50; supplemental data from a previous healthy control sample) for anger, happiness, sadness, fear, disgust, and neutrality recognition. The PTSD group and EE group had increased accuracy for recognition of all emotions compared to the HC control group. Pairwise comparisons displayed the PTSD group was significantly less accurate in identifying sadness than the EE group, however this result was no longer significant when depression and anxiety ratings were controlled. The PTSD and EE groups more were likely to misidentify neutral expressions as angry and disgusted compared to the HC group. The HC group also misinterpreted neutral expressions as happy.

Pfaltz et al. (2019) aimed to determine if negative interpretation of neutral faces occurs in individuals with PTSD (*n* = 39), compared to a trauma-exposed control group (TC; *n* = 44), and a no-trauma exposed control group (HC; *n* = 35) for recognition of joy, pride, sadness, fear, anger, disgust, contempt, embarrassment, surprise, or neutrality in video clips. The TC group had experienced at least one traumatic event (by DSM-5 criteria) but did not meet criteria for current or past PTSD. The HC had never experienced a traumatic event according to DSM-5 criteria. Results demonstrated there was not a significant effect of diagnostic group on emotion recognition accuracy or misinterpretation. Analyses determined childhood sexual abuse was the best predictor for recognition accuracy. Further analyses found higher levels of sexual abuse was linked to worse accuracy for neutral faces and higher levels of childhood maltreatment was linked to misinterpreting neutral expressions as contempt and anger.

Poljac et al. (2011) also used video clips of an actor expressing an emotion (i.e., anger, disgust, fear, happiness, sadness, or surprise) to understand emotion recognition patterns in a PTSD group (*n* = 20) and a control group (*n* = 20). Both PTSD and control groups were war veterans who had experienced prolonged traumatic events during the Bosnian war. Emotion recognition analyses indicated the PTSD group had reduced recognition of fear and sadness compared to the control group. Additionally, the PTSD required greater intensity of fear and sadness to accurately identify it. Covariate analyses revealed depression did not influence results.

#### Body dysmorphic disorder (*n* = 6)

Buhlmann et al. (2004) investigated emotion recognition trends within BDD (*n* = 20), OCD (*n* = 20), and a control sample (*n* = 20) on recognition of anger, disgust, happiness, sadness, fear, surprised, and neutrality. BDD group was significantly less accurate for overall, neutral, and disgust recognition compared to the control group. There were no significant group differences between the OCD and BDD groups or the OCD and control groups. BDD group also misinterpreted disgust as anger and interpreted more faces as anger than the control group.

A further study conducted by Buhlmann et al. (2006) studied emotion recognition within BDD (*n* = 18) and a control group (*n* = 18) on the recognition of anger, disgust, surprise, and neutrality for two different scenarios (i.e., self-referent and other-referent). Self-referent scenarios asked participants to imagine the emotion stimuli were faces looking at them (rather than looking at other people) and per this definition, this scenario appears to be the most congruent with other reviewed tasks. The BDD group was less accurate in overall and neutral recognition in self-referent scenarios and there was no significant group difference for other-referent scenarios. The BDD group significantly misinterpreted more neutral expressions as contemptuous and as angry than controls. There was a non-significant trend that the BDD group misinterpreted more neutral faces as disgust. When subgroup analyses were performed without individuals with SAD or major depressive disorder (MDD), respectively, results showed comorbid SAD did not affect patterns, however, group differences were no longer significant without individuals with MDD.

Buhlmann et al. (2011) continued study of emotion recognition patterns in comparing BDD (*n* = 34), dermatological-conditions (DC; *n* = 34), and a control sample (*n* = 34) in the recognition of anger, disgust, happiness, neutrality, sadness, fear, and surprise. The DC group did not meet criteria for current or past BDD. Analyses demonstrated there were no group differences in overall emotion recognition. However, the BDD group was significantly less accurate for neutral expressions than the control and DC groups. The BDD group misinterpreted neutral expressions as disgust more often than the control or DC groups, though after Bonferroni corrections, this trend was no longer significant.

Grace et al. (2019) studied a BDD group (*n* = 19) and control group (*n* = 21) on the recognition of happiness, sadness, fear, anger, and neutrality. BDD was linked to significantly worse recognition overall and of anger, neutrality, and sadness compared to control group. OCD symptoms, depression, and anxiety did not influence results as revealed by covariate analyses.

Jefferies et al. (2012) compared among individuals with BDD (*n* = 12) and controls (*n* = 16) on the recognition of happiness, surprise, fear, sadness, disgust, and anger. Analyses demonstrated the BDD group had significantly worse recognition of fear than control group. When grouping the emotions, it was found the BDD group recognized *no-threat* emotions (i.e., happiness, sadness, surprise) better than *threat* emotions (i.e., fear, anger, disgust). Covariate analyses found anxiety and depression did not influence pattern of results.

Toh et al. (2015) examined emotion identification within BDD (*n* = 21), OCD (*n* = 19), and a control sample (*n* = 21) for anger, disgust, fear, happiness, sadness, surprise, neutrality. The BDD group had overall reduced recognition of emotional expressions compared to the control and OCD groups, but there was not a significant interaction between group and facial affect. Moreover, the BDD group misinterpreted more faces as angry relative to OCD and control groups, highlighting an anger recognition bias.

#### Trichotillomania and skin picking disorder (*n* = 1)

Aydin et al. (2022) looked at recognition patterns for happiness, surprise, fear, sadness, anger, disgust, and neutrality in TTM (*n* = 30), SPD (*n* = 40), and controls (*n* = 30). After depression, anxiety, and alexithymia were controlled, SPD group had worse overall and disgust accuracies compared to control group. Analyses without covariation were not reported. There were no significant differences between the TTM and SPD groups or TTM and control group.

### Literature critique

Potential conclusions and patterns to be found in the considered literature must be evaluated within the limitations of the present studies as well as their strengths. As such, the studies included in this review will be appraised based on methodology, features of the emotion recognition task, and statistical and analytical procedures.

#### Sample characteristics

A potential threat to internal validity is the study sample size, generalizability, and thus possible analytical consequences. The total participants in each study ranged from 28 to 167. However, given the breadth of disorders covered in the present review, a source of strength is that the literature on PD, PTSD, BDD, and SPD/TTM have at least one study within the field with a larger sample size (e.g., >50). Therefore, GAD is the only disorder that may be more at risk to non-generalizable conclusions due to sample size.

Studies differed on the amount of demographic data provided and the variability in the sample. Seven studies reported there were no statistical difference between clinical and control groups regarding education level, removing a possible threat to internal validity (Aydin et al., 2022; Buhlmann et al., 2004, 2011; Grace et al., 2019; Jefferies et al., 2012; Poljac et al., 2011; Reinecke et al., 2011). Buhlmann et al. (2006) and Toh et al. (2015) had significant differences between education level in their clinical and control samples, which may contribute noise to observed group differences. All studies provided gender information for their samples, however nine studies included total samples that had a 66% proportion of women or larger (see Table 1). The greater proportion of female participants may represent an issue of oversampling in these studies, though it may also potentially represent a higher proportion of female involvement in research participation generally. Dickinson et al. (2012) reported female participants are overrepresented in undergraduate participant pools.

One clear weakness in the field is the reporting of ethnic and racial group information regarding study participants. Only one study, Buhlmann et al. (2011), reported racial group demographic information regarding their participants. As the studies reviewed were based in countries around the world and as recruitment sources were often local clinics or community volunteers, it can be reasonably assumed there is a diversity in nationalities represented, though generalizability of findings cannot be guaranteed.

Another consideration to internal validity is the presence of comorbidities in the samples (see Table 3). In samples that excluded comorbid disorders (*n* = 4), study results and conclusions are strengthened for the specific clinical population. However, in the real-world, many individuals with an anxiety, obsessive-compulsive and related, or trauma- and stressor-related disorder do have comorbid diagnoses, so excluding these individuals may also limit generalizability of findings into community or broader samples (Salcedo, 2018). As with many clinical investigations, comorbid diagnoses must be considered and accounted for in statistical analyses; such procedures are reviewed in Covariate Analyses section.

An important factor to consider is medication and/or treatment of the clinical population if it is not the target of the study. Treatment and medication whose primary aim is to relieve distressing symptoms could also ameliorate behavioral patterns which are the subject of research. Seven of the current studies enrolled participants that were receiving psychiatric treatment for mood concerns (see Table 1). Buhlmann et al. (2006) reported that over one half of their clinical BDD sample was receiving cognitive-behavioral therapy at the time of study participation. Medications and treatment aim to have behavioral impacts related to clinical distress and thus individuals participating in such treatments may have differential performance in emotion recognition tasks (more similar to control groups). See Covariate Analyses for a review of statistical methods to address such concerns.

A key strength of the reviewed articles lies in the diagnostic procedure for the clinical and control samples. Three studies used a clinical/psychiatrist interview to confirm clinical diagnoses (Bell et al., 2017; Reinecke et al., 2011; Wang et al., 2013). Clinical and control group criteria was confirmed using the SCID diagnostic interview for DSM, DSM criteria (DSM-4, DSM-4-TR, or DSM-5), or Mini-International Neuropsychiatric Interview (MINI) for the remaining 12 studies. The MINI and SCID are validated diagnostic tools to assess DSM criteria and therefore enhance internal validity of those studies (Osório et al., 2019; Sheehan et al., 1998).

Another strength of the literature is the inclusion of a control sample. All studies included a control group sample that, at a minimum, did not meet diagnostic criteria for any axis 1 psychiatric disorders. Of note, the control group in Poljac et al. (2011) were war veterans with trauma exposure and therefore results are limited by the fact there is not a true comparison group without any trauma experience in this study. Additionally, three studies included an additional comparison group which had overlapping experiences (e.g., traumatic experience, skin condition) with the clinical group but did not have the diagnosis of interest (Bell et al., 2017; Buhlmann et al., 2011; Pfaltz et al., 2019). In these cases, the study results are bolstered in comparing all three groups and determining if any patterns are specific for the clinical diagnosis (rather than a shared experienced) and thus removing more potentially confounding variables for the emotion recognition patterns found.

Another consideration in the evaluating the representativeness of the sample and thus the external validity of the conclusions is the recruitment methods and sampling characteristics. Only one study did not report sampling methods (Aydin et al., 2022). All remaining studies deployed convenience sampling for their participants. Furthermore, given the lack of racial group demographic data, it is impossible to ascertain the effects of convenience sampling in terms of racial/ethnic group diversity in the study participants.

Additionally, 10 studies recruited their clinical samples from outpatient treatment centers and Poljac et al. (2011) recruited from a PTSD support group for war veterans (see Table 1). With these recruitment sources, individuals have sought treatment and may be receiving ongoing care from the treatment facility. As mentioned above, possible care from treatment centers and support groups may have led to symptom reduction and therefore impact behavioral results in recognition task and could also lead to a sampling bias in clinical severity representation.

#### Emotion recognition task considerations

The studies varied in the amount of time each emotional stimulus was presented to participants; there was a reported range of 200–15,000 ms. In studies with a shorter stimulus duration, the tasks are measuring an immediate, possibly unconscious, perception of the emotional stimulus. On the other hand, studies with a longer stimulus duration may be evaluating participants' conscious evaluation of the stimuli. Procedures also differed in labeling procedures. Three studies reported participants labeled the emotional face after the stimulus was removed while seven reported participants labeled the stimulus during presentation (see Table 2); studies may therefore differ in testing the interpretation of a *memory* of the emotional stimulus rather than the direct interpretation. Memory biases for certain emotions or valences (i.e., negative interpretation bias) may be driving results. As such, the timing of labeling interpretation may be a source of issue in interpreting study results across disorders.

On the other hand, one strength regarding task construct validity in comparing study results within and across disorders is the overlapping use of emotional stimuli. Emotional stimuli from Ekman and Friesen (1976) were used in 10 studies as source material (Aydin et al., 2022; Bell et al., 2017; Buhlmann et al., 2004, 2006, 2011; Grace et al., 2019; Jefferies et al., 2012; Palm et al., 2011; Reinecke et al., 2011; Toh et al., 2015). Though it cannot be confirmed these studies used the exact same photos for their task stimuli, using the same source material enhances the comparability between study results. Several studies used emotional stimuli that were intentionally more ethnically similar to the study population (Cai et al., 2012; Pfaltz et al., 2019; Wang et al., 2013). Jefferies et al. (2012) and Kessler et al. (2007) reported their emotion recognition tasks had been validated in previous work (Cronbach's alpha = 0.77; Kessler et al., 2007). None of the other studies reported the validity of the used emotion recognition task.

Review conclusions are bolstered by the consideration that 14 of the 15 studies used emotional stimuli from multiple actors (i.e., different people's faces); only Bell et al. (2017) did not report this statistic. Using multiple emotional faces increases the generalizability of study results; it increases the confidence that study results were not particular to a specific person (stimulus), but rather trends in the emotional expression across people. There was also variability in the number of trials (i.e., 24–300) within the emotion recognition task (see Table 2). Though more trials increase the validity of the acquired data, it may also introduce practice effects. It is possible that individuals who completed more trials answered subsequent trials more habitually and decreased cognitive processing. All studies had repeated trials for each emotional stimulus, increasing reliability of results per each stimulus, and thus each emotion.

One major strength of this field is the breadth of emotions used in the recognition tasks; all studies evaluated at least four emotions (see Table 2). Overall, anger, happiness, sadness, fear, surprise, disgust, and neutrality were all examined in at least 11 studies. All of the studies investigating patterns with PTSD tested the recognition of anger, happiness, sadness, fear, disgust, and neutral faces. Within BDD, all studies looked at anger, and most (five of six) examined happiness, sadness, fear, and disgust. Given BDD also had the most studies included in the present review, conclusions regarding this disorder can be drawn from a variety of sources. All of the PD studies examined recognition of anger, happiness, sadness, and fear. Overlap in emotions allows for better comparison and differentiation between studies and emotions.

It should also be noted that half of the BDD studies have the same primary author and therefore have a narrow stimulus sampling, proving to be a threat to external validity (Buhlmann et al., 2004, 2006, 2011). Two of these studies were conducted in the same geographic location and published within 2 years of each other; it is possible there is overlap in participants between them (Buhlmann et al., 2004, 2006). If there was any participant overlap (i.e., individuals who participated in both studies), this was not reported. Such overlap would decrease the variability in participant data in the field.

Pfaltz et al. (2019) and Poljac et al. (2011) were the only studies to use video progressions for their emotional stimuli material. Therefore, the PTSD literature discussed relies more heavily on dynamic stimuli than static images. It is possible a video progression of emotion expression would lead to differential recognition accuracies than static images. The person may be better able to determine the key facial features in the emotion expressed (by their gradual change) and thus identify the expressed emotion more easily. However, it is also possible that viewing a video expression of an emotion may include other distractor variables and lead to more difficulty for participants to identify the correct emotion.

The other 13 studies reviewed used static photos as emotional stimuli. Within these studies, five of them also included morphed emotional stimuli (computer generated images; see Table 2). These images are anchored in a real person's facial expressions (i.e., neutral and angry) and then computer software is used to create facial expressions that represent the transition from neutrality to emotion. None of the BDD studies used morphed images in their tasks and thus participants only identified natural emotional faces. The PD and PTSD literature includes both natural and morphed image use. It is possible that these morphed emotional faces represent a less ecologically valid form of the emotion recognition task. There is no current research investigating difference in performance between emotion recognition tasks with natural stimuli and computerized stimuli. Therefore, the conclusions from the BDD studies may be more ecologically valid.

All studies used a within-subject design for their emotion recognition task. As such, all subjects were tested on all emotional stimuli and included in analyses. Within-subject design decreases the risk of errors resulting from differences between participants, reducing error variance. It also increases the statistical power of analyses. However, potential risks to internal validity with such methodology are practice effects and order effects for the task. To combat possible order effects, nine studies used a randomized design in the emotion recognition task. Six studies did not report task randomization (see Table 2). Randomization of emotional stimuli decreases potential order effects, carryover effects, and fatigue effects of participants which may impact stimulus labeling response and threaten internal validity and reliability.

When examining the potential impact of task parameters on study results, it was determined that procedural decisions such as labeling procedure, length of stimulus, morphed vs. natural photos, and number of trials did not influence pattern of results.

#### Statistical and analytical considerations

##### Effect size

Effect sizes aid in the interpretation of study results by representing the magnitude of study effects irrespective of sample size (Fritz et al., 2012). In the current review, five studies reported effect sizes for relevant statistics (Buhlmann et al., 2006, 2011; Cai et al., 2012; Grace et al., 2019; Reinecke et al., 2011). Jefferies et al. (2012) and Toh et al. (2015) reported effect sizes for some analyses, but not all. Of the remaining articles, effect sizes were calculated based on statistics provided but were unable to be calculated for Poljac et al. (2011) due to insufficient data provided in the text.

Within GAD, Palm et al. (2011) a large effect size for impaired recognition of sadness was found. Within PD groups compared to control groups, medium effects were found for impaired recognition of disgust, fear, sadness, anger, and overall emotion recognition (Cai et al., 2012; Kessler et al., 2007). However, medium effects were enhanced recognition of sadness and overall emotion recognition were also found (Reinecke et al., 2011; Wang et al., 2013). A large effect for enhanced recognition of surprise was also found (Cai et al., 2012).

In comparing PTSD to non-trauma-exposed individuals, large effects for enhanced recognition of neutral, angry, happy, sad, fearful, and disgusted faces were found (Bell et al., 2017). Of note, Bell et al. (2017) also found a large effect of enhanced emotion recognition between the EE and HC groups. Furthermore, there was a large effect for decreased recognition of sadness between the PTSD and EE groups (Bell et al., 2017). Effect sizes were unable to be calculated for Poljac et al. (2011) due to insufficient data provided in the text.

For BDD groups, large effects were related to decreased recognition of neutral, disgusted, angry, sad, and fearful emotional faces (Buhlmann et al., 2004, 2006; Grace et al., 2019; Jefferies et al., 2012). Medium and large effects were linked to overall worse emotion recognition (Buhlmann et al., 2004, 2006; Grace et al., 2019; Toh et al., 2015). Toh et al. (2015) found medium effect sizes for decreased recognition of anger and sadness and a small effect for fearful faces. Within SPD, large effects were found for reduced recognition of disgust and overall emotion recognition in SPD compared to controls (Aydin et al., 2022).

##### Power

To further evaluate study quality and strength of conclusions, *post-hoc* power analyses were conducted for articles that provided adequate information. Using recommended criteria of a power level of at least 0.80 per Cohen (1992), Reinecke et al. (2011) and Wang et al. (2013) were insufficiently powered for analyses. Three additional studies were insufficiently powered for at least one of their emotion recognition analyses (disgust accuracy in Cai et al., 2012; overall emotion accuracy in Grace et al., 2019; fear and sadness accuracy in Toh et al., 2015). Therefore, results from these studies regarding these analyses must be reviewed with caution. The remaining studies reviewed were sufficiently powered for their analyses.

Given the examination of multiple emotions, studies used ANOVAs or Mann Whitney U tests to determine group differences. Such analyses run the risk of an increased type 1 error due to multiple statistical tests. Eleven of the current studies used *post-hoc* adjustments (e.g., Bonferroni corrections, etc.) to reduce the risk of this error; as Bell et al. (2017), Jefferies et al. (2012), Poljac et al. (2011), and Reinecke et al. (2011) did not report such statistical corrections, there is an increased risk for incorrectly concluding significant group differences in these studies.

##### Misinterpretation analyses

A further strength in this field is the presence of misinterpretation analyses, which serve to clarify if a particular emotion was more often identified as a different emotion (rather than simply incorrectly identified). Eight studies reported such analyses (Bell et al., 2017; Buhlmann et al., 2004, 2006, 2011; Kessler et al., 2007; Pfaltz et al., 2019; Reinecke et al., 2011; Toh et al., 2015). Effect sizes and *post-hoc* power could not be calculated for Pfaltz et al. (2019) and Toh et al. (2015) as sufficient information was not provided. Within PD, there was contradictory evidence for an anger recognition bias (interpret other emotions as anger) as Kessler et al. (2007) reported a medium effect size, but Reinecke et al. (2011) found no group difference for misinterpretation toward anger. Though, using *post-hoc* power guidelines described previously, Kessler et al. (2007) was insufficiently for this analysis. The PTSD literature demonstrated support for trauma-exposed groups misinterpreting neutral expressions as anger with a large effect size in Bell et al. (2017) (Pfaltz et al., 2019). BDD clinical groups misinterpreted more expressions as angry (Buhlmann et al., 2004, 2006; Toh et al., 2015) with sufficiently powered, large effect sizes linked to the specific misinterpretation of disgust faces as angry and neutral faces as contemptuous (Buhlmann et al., 2004, 2006).

##### Covariate analyses

Seven studies analyzed the potential impact of comorbid mental illness in the clinical population with covariate analyses, and depression was evaluated as a covariate for all of these studies (see Table 3). Depression and anxiety were found to have potentially impacted recognition patterns for PD (Kessler et al., 2007). Within BDD and PTSD there were conflicting results on the impact of depression; some studies did not find depression influenced recognition patterns (Grace et al., 2019; Jefferies et al., 2012; Poljac et al., 2011) while other studies did find an impact (Bell et al., 2017; Buhlmann et al., 2006). Aydin et al. (2022) controlled for alexithymia, depression, and anxiety in analyses and found there was still a significant difference between the SPD and control group for recognition of disgust and overall recognition. Comorbid social anxiety, anxiety, and OCD symptoms in BDD was not found to influence results (Buhlmann et al., 2006; Grace et al., 2019). Only one study looked at the potential impact of psychotropic medication and found Escitalopram and Paroxetine use did not impact emotion recognition patterns nor was there a significant connection between medication dosage or duration and emotion accuracy in PD (Wang et al., 2013).

## Discussion

### Synthesis of findings

The present systematic review sought to explicate potential patterns within recognition of emotions and among anxiety, obsessive-compulsive and related, and trauma- and stressor-related disorders. All studies reviewed examined potential differences between a clinical sample and a control sample on an emotion recognition task. All emotion recognition tasks looked at recognition patterns among at least four distinct emotions (including neutrality), with a median of seven emotions studied. Potential group differences must be reviewed relative to individual study strengths and limitations as well as those among all reviewed studies for each disorder.

#### Generalized anxiety disorder

Patterns related to GAD can only be compiled from a single study. Palm et al. (2011) GAD was linked to significantly worse recognition of sadness with a sufficiently powered large effect. Study results are potentially strengthened by the exclusion of participants with current MDD, as covariate analyses conducted in other disorders identified depression as a potential confounding variable in recognition patterns. Palm et al. (2011) did not conduct analyses to determine impact of comorbid anxiety disorders. As such, Palm et al. (2011) introduces preliminary evidence for a bias of misidentification for sadness in GAD, but this claim needs to be further examined in future research with adequate attention to statistical control of potential confounding variables (i.e., comorbid disorders, treatment).

#### Panic disorder

Literature supported a link between PD and worse recognition of disgust, fear, sadness, anger, and overall emotion accuracy (Cai et al., 2012; Kessler et al., 2007). Contrasting results from Reinecke et al. (2011) and Wang et al. (2013) do not detract from this finding due to increased risk of type 1 error, insufficient power, and clinician-rendered group assignment rather than a standardized, validated diagnostic tool (e.g., SCID, MINI). It is possible this diagnostic method may have led to more variability in the clinical sample, which could contribute to noise in the results. Additionally, the literature included studies in which participants labeled stimuli during the task and following the task, so it is unclear if tasks are assessing emotion recognition or memory. The previous mini-review appears to agree with trends found is this review, as Bottinelli et al. (2021) supplicated PD was linked to worse overall emotion recognition, specifically negative emotions. Of note, Cai et al. (2012) and Kessler et al. (2007) were included in the mini-review.

While they agreed on worse recognition patterns in the PD group, Cai et al. (2012) and Kessler et al. (2007) did not overlap on any conclusions for specific emotions they both examined. While neither of these studies included clinical populations with other anxiety disorders or MDD, Kessler et al. (2007) found anxiety and depression ratings may have impacted results. While mood measures may explain some variability in recognition patterns, it does not necessarily mean results are not also explained by PD as depressive and anxiety symptoms could be tied to the severity of PD presentation. It may also indicate more severe PD presentation leads to decreased emotion recognition. Given the lack of consensus on covariate analyses, measures of clinical severity, and trends in recognition patterns, specific emotion recognition accuracies for PD cannot be reliably concluded and need further investigation and replication. The PD field would also benefit from increased misinterpretation analyses to further tease out possible patterns as current analyses were underpowered.

#### Posttraumatic stress disorder

Similar to PD, the PTSD literature included studies in which participants labeled stimuli both during the task and following the task, so it is unclear if tasks are assessing emotion recognition or memory. Results across the PTSD literature may suggest trauma-experience and type of trauma is better related to emotion recognition performance, rather than PTSD diagnosis. Earthquake-PTSD and earthquake-exposed groups performed similarly on recognition tasks in Bell et al. (2017) compared to a non-exposed control group. In evaluating multiple sources of trauma, Pfaltz et al. (2019) did not find significant group differences between PTSD and a control group. However, in categorizing groups by types of traumas, it found higher levels of childhood sexual and emotion abuse were linked to decreased accuracy of neutral faces and misinterpretation of neutral faces (as contempt and anger). Furthermore, childhood sexual abuse was identified as the most relevant predictor of emotion recognition accuracy. There was initial evidence for decreased recognition of sadness linked to PTSD diagnosis compared to a trauma-exposed group (Bell et al., 2017; Poljac et al., 2011); but this result must be reviewed with caution. Depression and anxiety measures were found to influence this result in Bell et al. (2017) and while depression did not affect results in Poljac et al. (2011), *post-hoc* effect sizes and power analyses could not be calculated. Both studies also had an increased risk of type 1 error.

In accumulating all evidence, the literature proposes emotion recognition patterns in PTSD are better characterized by the type of trauma experienced rather than diagnostic characterization, though this needs further replication. There is preliminary evidence that PTSD diagnosis is associated with decreased recognition of sadness, however this finding needs further investigation with trauma type and depression/mood measures and robust statistical considerations. Furthermore, there is initial evidence that trauma-exposure may be associated with misinterpretation of neutral expressions as angry (Bell et al., 2017; Pfaltz et al., 2019).

#### Body dysmorphic disorder

Similar to the other disorders discussed, the BDD literature included studies in which participants labeled stimuli both during the task and following the task, so it is unclear if tasks are assessing emotion recognition or memory. Decreased recognition of neutrality, disgust, sadness, and fear were all demonstrated in the BDD literature (Buhlmann et al., 2004, 2006, 2011; Grace et al., 2019; Jefferies et al., 2012). Decreased recognition of neutral expressions was the most supported in the literature with sufficiently powered analyses and samples with comorbidities. But there were conflicting results on the potential impact of depression: this result was no longer supported when MDD participants were removed in Buhlmann et al. (2006), but it remained when depression and anxiety were controlled in Grace et al. (2019) and Jefferies et al. (2012). However, Jefferies et al. (2012) possibly had an increased risk of type 1 error. Misinterpretation biases supported an anger interpretation bias, in which non-anger expressions were more often misidentified as anger in BDD clinical samples (Buhlmann et al., 2004, 2006; Toh et al., 2015).

#### Trichotillomania

The limited literature proposes preliminary evidence that TTM does not influence emotion recognition patterns (Aydin et al., 2022). There were no group differences in performance between TTM, SPD, and control groups when depression ratings, anxiety ratings, and rates of alexithymia were controlled in in statistical analyses. Analyses without these covariates were not reported. The conclusions are strengthened by the large sample size in Aydin et al. (2022) but must be considered with caution as they are based on a singular study.

#### Skin picking disorder

The literature suggests SPD is linked to decreased disgust and overall emotion recognition (Aydin et al., 2022). After potential impacts of depression, anxiety, and alexithymia were controlled for, the SPD group had significantly worse disgust recognition and overall recognition than the control group. The SPD group did not perform differently than the TTM group, a finding that supports the need for further replication in research. Future research should report comorbid diagnoses present in samples and adjust analyses accordingly. As results for SPD are based on a singular study, emotion recognition patterns are thus only initially suggested and require further evidence from the field.

### Limitations and future directions

Several key limitations appeared throughout the literature. Comorbidity in the included studies is both a strength (increases external validity) and a weakness (decreases internal validity; especially when comorbidity was not assessed or reported). Information regarding current medication treatment and current therapeutic treatment would also be beneficial and relevant in considering behaviors (e.g., emotion recognition) related to clinical diagnoses.

With increased clinical utility in including participants with comorbidities, such comorbidities should be examined further in statistical analyses. Of the studies that examined depression as a covariate, there was conflicting impact of depression on patterns. Breteler et al. (2021) found comorbid depression was linked to more severe symptoms pre- and post-treatment for anxiety, OCD, and PTSD. In the current review, it is possible that more clinical severe presentations of a disorder were linked to increased depressive symptoms, but this needs further investigation.

There was a lack of racial group demographic data reported for study participants as well as face stimuli. Racial hostility biases have been demonstrated in previous literature and may demonstrate potential confounding variables in the emotion recognition tasks reviewed. Hugenberg and Bodenhausen (2003) found White participants high in implicit racial prejudice identified anger quicker in Black men compared to White men in an emotional recognition task; this research proposes racial prejudice can impact emotion recognition task results and patterns and thus future research should report such demographics.

In the current systematic review, only Jefferies et al. (2012) and Kessler et al. (2007) reported validation for their emotion recognition task. Future studies should take care to include emotion labeling procedures (vs. memory tasks), thereby better assessing interpretation versus memory. It would be beneficial for future research to examine the impact of methodological differences in emotion recognition tasks (e.g., stimulus durations, labeling procedures). Research would also benefit from comparing morphed to natural stimuli, as well as video to static stimuli. Research within the field would also be strengthened by including power analyses for result interpretation. Future research could also consider implicit perception of facial emotions.

While the fields of PD, PTSD, and BDD are strengthened by multiple studies, there is limited research on emotion recognition within GAD, TTM, and SPD. Thus, claims regarding these disorders are provided with caution as they are solely based on single studies. PTSD literature relies more heavily on dynamic emotional stimuli rather than static, but there is not yet research comparing recognition between types of stimuli.

There is a potential risk of publication bias in the current review, given only studies that were accepted and published were considered. Therefore, the present issues that serve as barriers in publication of studies (e.g., non-significant results) could have impacted the current review. Furthermore, certain procedures relevant to this review (e.g., literature search, screen, data extraction) were completed solely by the first author. As such, it is possible that relevant studies and germane data were missed. While this may introduce potential bias, this concern is mitigated and we have enhanced transparency by following PRISMA reporting guidelines. Given 15 studies were evaluated across six clinical disorders, we are unable to conduct a meta-analysis on the provided data. Future research should evaluate emotion recognition trends in anxiety and related disorder and once there have been more published studies, a meta-analysis would be beneficial for the field.

A strength of the present review is its adherence to PRISMA guidelines, supporting a well-researched and adequate search of available literature (Page et al., 2021). As all of the studies applied a within-subject procedure, results are protected against influence from potential confounding variables related to certain participant characteristics. All studies reviewed at least four different emotions in the task, therefore providing analyses on multiple recognitions for comparisons within a study and across studies. Both large and medium effect sizes were demonstrated for applicable significant differences in group performance. Furthermore, a majority of studies were appropriately powered for their statistical analyses.

### Conclusion

The aim of this review was to identify emotion recognition patterns among anxiety, obsessive-compulsive and related, and trauma- and stressor-related disorders. Results suggest such disorders are associated with decreased emotion recognition accuracy. Previous SAD and OCD reviews suggest clinical samples had decreased emotion recognition (Daros et al., 2014; Lacombe et al., 2023). In the present review, decreased recognition of sadness was indicated in GAD, PD, PTSD, and BDD. An anger interpretation bias was preliminarily indicated in PD, BDD, and certain trauma types. These patterns align with theory linking emotion recognition to clinical disorders through a fear of negative evaluation (Buhlmann et al., 2006). If an individual tends to interpret anger in others' expressions, they will perceive it is directed at them, increasing their distress and symptom experience. A deficit in sadness recognition may similarly represent a fear of negative evaluation that maintains symptoms (Grace et al., 2019). Given the similarity of these patterns across disorders, it is possible an anger interpretation bias and reduced sadness accuracy is characteristic for anxiety and related disorders, though this cannot be strongly concluded without further replication and bolstering of study reporting, procedures, and analytic techniques.

The lack of replicated results within robust studies may suggest specific emotion recognition patterns in anxiety and related disorder are not best characterized by diagnoses. While overall emotion recognition deficits were observed, it is possible that specific emotion recognition is better understood by other frameworks, such as transdiagnostic factors like intolerance of uncertainty (Yigman and Fidan, 2021) and anxiety sensitivity (Fairholme et al., 2012). The PTSD literature indicates types of traumas must be studied and considered. As depression proved to inconsistently impact results in PD, PTSD, and BDD, it also requires further investigation. Clinical severity of diagnoses must be researched and statistically considered for its impact as well as its connection to depression and mood measures. Such additional models may better explain variability and patterns in emotion recognition deficits and provide next steps for research and treatment for anxiety and related disorders.

## Data Availability

The raw data supporting the conclusions of this article will be made available by the authors, without undue reservation.

## Footnotes

^1^Task design information was reported from source material, Harmer et al. (2001).

## Appendix A

Keywords used for all databases

*Studies with diagnoses characterized as anxiety, obsessive-compulsive and related, and trauma- and stressor-related disorders per DSM-5 were searched for in databases. Search terms that yielded studies included in the current review after screening and eligibility process are presented below*.

(emotion recognition OR emotion perception OR emotion identification) AND (generalized anxiety disorder OR gad)

(emotion recognition OR emotion perception OR emotion identification) AND (panic disorder)

(emotion recognition OR emotion perception OR emotion identification) AND (ptsd OR post traumatic stress disorder OR posttraumatic stress disorder OR post-traumatic stress disorder)

(emotion recognition OR emotion perception OR emotion identification) AND (body dysmorphic disorder OR bdd)

(emotion recognition OR emotion perception OR emotion identification) AND (trichotillomania OR hair pulling disorder)

(emotion recognition OR emotion perception OR emotion identification) AND (skin picking disorder OR excoriation disorder).
